# Comparison of dialysis dose through real-time Kt/V by ultraviolet absorbance of spent dialysate, single-pool Daugirdas II, and Kt/BSA according to sex and age

**DOI:** 10.1590/2175-8239-JBN-2020-0081

**Published:** 2020-12-07

**Authors:** Mauro Sergio Martins Marrocos, Christine Nastri Castro, Wilder Araujo Barbosa, Andressa Monteiro Sizo, Fernanda Teles Rodrigues, Rosemary Alves de Lima, Sandra Maria Rodrigues

**Affiliations:** 1 Hospital do Servidor Público do Estado de São Paulo, São Paulo, SP, Brasil.

**Keywords:** Renal Dialysis, Matched-Pair Analysis, Body Surface Area, Online Systems, Diálise Renal, Análise por Pareamento, Superfície Corporal, Sistemas On-Line

## Abstract

**Background:**

Kt/V OnLine (Kt/VOL) avoids inaccuracies associated with the estimation of urea volume distribution (V). The study aimed to compare Kt/VOL, Kt/V Daugirdas II, and Kt/BSA according to sex and age.

**Methods:**

Urea volume distribution and body surface area were obtained by Watson and Haycock formulas in 47 patients. V/BSA was considered as a conversion factor from Kt/V to Kt/BSA. Dry weight was determined before the study. Kt/VOL was obtained on DIALOG machines.

**Results:**

Pearson correlation between Kt/VOL vs Kt/VII and Kt/VOL vs Kt/BSA was significant for males (r = 0.446, P = 0.012 and r = -0.476 P = 0.007) and individuals < 65 years (0.457, P = 0.019 and -0.549 P = 0.004), but not for females and individuals ≥ 65 years. V/BSA between individuals < 65 and individuals ≥ 65 years were 18.28 ± 0.15 and 18.18 ± 0.16 P = 0.000). No agreement between Kt/VII vs Kt/BSA. Men and individuals > 65 years received a larger dialysis dose than, respectively, females and individuals < 65 years, in the comparison between Kt/VOL versus Kt/VII. V/BSA ratios among men and women were respectively 18.29 ± 0.13 and 18.12 ± 0.15 P = 0.000.

**Conclusions:**

Kt/VOL allows recognition of real-time dose regardless of sex and age.

## RESUMO

**Introdução:**

O Kt/V OnLine (Kt/VOL) evita imprecisões associadas à estimativa da distribuição do volume de uréia (V). O estudo teve como objetivo comparar Kt/VOL, Kt/V Daugirdas II e Kt/BSA de acordo com sexo e idade.

**Métodos:**

A distribuição do volume de uréia e área de superfície corporal foram obtidas pelas fórmulas de Watson e Haycock em 47 pacientes. V/BSA foi considerado um fator de conversão de Kt/V para Kt/BSA. O peso seco foi determinado antes do estudo. Kt/VOL foi obtido através de máquinas DIALOG.

**Resultados:**

A correlação de Pearson entre Kt/VOL vs Kt/VII e Kt/VOL vs Kt/BSA foi significativa para os homens (r = 0,446, P = 0,012 e r = -0,476 P = 0,007) e indivíduos < 65 anos (0,457, P = 0,019 e -0,549 P = 0,004), mas não para mulheres e indivíduos ≥ 65 anos. A V/BSA entre indivíduos <65 e indivíduos ≥ 65 anos foi 18,28 ± 0,15 e 18,18 ± 0,16 P = 0,000). Sem concordância entre Kt/VII vs Kt/BSA. Homens e indivíduos > 65 anos receberam maior dose de diálise do que, mulheres e indivíduos <65 anos, respectivamente, na comparação entre Kt/VOL versus Kt/VII. As razões V/BSA entre homens e mulheres foram, respectivamente, 18,29 ± 0,13 e 18,12 ± 0,15 P = 0,000.

**Conclusões:**

Kt/VOL permite o reconhecimento da dose em tempo real, independentemente do sexo e idade.

## Introduction

Dialysis adequacy is traditionally measured by monthly blood urea sampling and calculating the sessional Kt/V single pool (single urea compartment) (Kt/V_urea_). The KDOQI (Kidney Disease Outcomes Quality Initiative) recommends this as a measure of hemodialysis efficiency and indicates an adequate minimum dose of 1.2 (with a target value of 1.4)^1^. Although studies^2^^,^ 3 ^,^ 4 raise doubts regarding the validity of the Kt/V_urea_ to report or prescribe dialysis dosage, it is still the most widely used adequacy parameter in clinical practice.

However, higher doses of dialysis are associated with lower mortality among women, but not among men^5^. Keeping in mind that toxic uremia is more associated with the metabolic rate than to the volume of distribution of urea^6^^,^ 7, underweight patients treated with a Kt/V of 1.2 would receive an inadequate dose of dialysis. The basal metabolic rate and the production of uremic toxins can be proportionally higher in smaller people, determining the need for a higher dose of dialysis. Less muscle mass (and consequently less volume of distribution for soluble uremic toxins) and less fatty tissue (thus smaller ability to adsorb uremic toxins) are also cited as explanations. Besides, women have a higher percentage of body water than men^8^.

It is coherent to imagine that people with a higher basal metabolic rate will need a higher dose of dialysis. Body surface area (BSA) correlates better in both sexes with the basal metabolic rate and body composition than urea distribution volume (V)^9^. The standardization of the glomerular filtration rate by BSA is the usual practice. The normalization of dialyzer clearance by BSA appears to be a logical extrapolation. At the same time, the elderly have a lower metabolic rate, less body water volume, less muscle mass, but the influence of these particularities in determining the dialysis dose is not clear^10^.

The Kt/V OnLine (Kt/VOL) through the ultraviolet absorbance in the spent dialysate corrects the inaccuracies associated with the V estimation and is considered more reliable than the usual calculation by urea kinetics^11^. The study aimed to compare Kt/VOL with Kt/V urea Daugirdas II(Kt/VII) and Kt/BSA according to sex and age.

## Methods

This was a cross-sectional cohort study undertaken at the Hemodialysis Unit of the Hospital do Servidor Público Estadual de São Paulo (HSPE-SP) between July and November 2016. The data were obtained from laboratory exams and Kt/VOL results obtained from the B BRAUN Dialog^+^ HD machines with a real-time Kt/V determination by ultraviolet absorbance in spent dialysate, on the same day blood sampling occurred. The standard dialysate flow, in all machines, was 500 mL/ min. Due to the observational nature of the study, no additional blood samples were taken, benefitting from the regularly performed dialysis dose measurements with pre- and post-dialytic blood samples to obtain the Kt/VII dialysis dose. Simultaneously to the standard method, the dialysis dose was also determined by two different non-invasive methods: Kt/BSA and Kt/VOL.

The following data were collected to calculate the Kt/VII: dry weight (DW), weight gain between sessions, height, age, sex, blood flow, and hematocrit. The determination of DW followed methodology from the study *Dry-Weight Reduction in Hypertensive Hemodialysis Patients* (DRIP)^12^. An initial additional weight loss of 0.1 kg/10 kg body weight was prescribed per dialysis without increasing the time or frequency of dialysis. This further weight loss was combined with the ultrafiltration volume required to remove interdialytic weight gain to achieve the desired reduction in DW. If ultrafiltration was not tolerated based on symptoms and signs such as muscle cramps, need for excessive saline, or symptomatic hypotension, the additional prescribed weight loss was reduced by 50%. If ultrafiltration was still not tolerated, the extra weight loss was further reduced by 50% till even 0.2 kg incremental weight loss per dialysis was not tolerated. We considered that the patient was at his or her DW at this point. The volume of ultrafiltration was programmed not to exceed the limit of 10 mL/kg/hour of hemodialysis session as a routine. Changes in antihypertensive medication were performed when necessary. The collection of clinical and laboratory data was conducted in the month immediately following the determination of the DW.

The value of V was obtained using the Watson formula: 

$$\left(2\right)V\left(L\right)=2.447-\left(0.09156\;x\;A\right)+\left(0.1704\;x\;H\right)+\left(0.3362\;x\;W\right)\;\left(\mathrm{men}\right)$$

The value of BSA was obtained using the Haycock formula:

$$\left(3\right)V\left(L\right)=\;-2.097\;-\;\left(0.1069\;x\;H\right)\;+\;\left(0.2466\;x\;W\right)\;\left(\mathrm{women}\right),\;\mathrm{where}:\;A=\mathrm{age}\;\left(\mathrm{years}\right),\;H=\mathrm{height}\left(\mathrm{cm}\right),\;W=\mathrm{weight}\;\left(\mathrm{kg}\right).$$

$$\begin{array}{c} \left.4\right)\mathrm{BSA}\left(m^{2}\right)\;=\;W^{0.5378}\;x\;H^{0.3964}\;x\;0.024265,\;\mathrm{where}:\\ W=\mathrm{weight}\left(\mathrm{kg}\right)\;\mathrm{and}\;H=\mathrm{height}\;\left(\mathrm{cm}\right). \end{array}$$

The value of target Kt was obtained using the formula: 

$$\left(5\right)\;\mathrm{Kt}=1.2\;x\;V.$$

The V/BSA ratio was regarded as the Kt/V correction factor for Kt/BSA^7^.

Data were analyzed according to sex and age: male and female and group I (< 65 years and group II (≥ 65 years). We compared the Kt/VOL results with the Kt/VII results obtained from the regularly performed dialysis dose measurements with pre- and post-dialytic blood samples to get the dialysis dose (Daugirdas II formula)^13^. In the absence of a surface-area-normalized dialysis dose formula, we determined the V/BSA ratio of the groups^6^^,^ 7.

### Patient Selection

The study was planned to enroll all patients of the Hemodialysis Unit of the HSPE-SP that met the inclusion criteria: adult age, physical and mental ability to participate in the study, treatment with B BRAUN Dialog+ HD machine, and signing of the informed consent form. Exclusion criteria were patients with limb amputation or significant atrophy, which cannot be weighed, and weighing more than 100 kg. 

### Statistics

Results are reported as mean ± SD. Student’s t-test was used to compare continuous variables. Pearson’s correlation was used to assess the degree of relationship between metric variables and their significance. The level of significance was set at 5% or 0.05 for all tests. As this was a proof-of-concept study, it was necessary to achieve 80% power^14^. A detected change of 0.1 between the Kt/V pairs and an expected standard deviation of 0.2 demanded a sample size of 34. The Statistical Package for the Social Sciences version 23.0 (SPSS) program was used for the statistical calculations.

### Regulatory Aspects

The Hemodialysis Unit of the HSPE-SP follows applicable local legal and administrative regulations. The Ethics Committee of the HSPE-SP approved the study (CAAE 47877915.0.0000.5463).

## Results

**Figure 1:**
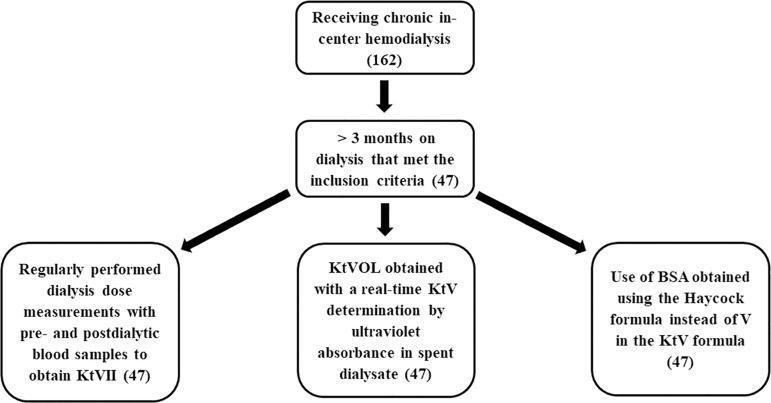
Patients flow diagram.

We evaluated the information of 47 patients, of whom 31(65.96%) were male and 16 (34.04%) female, with a mean age of 63.06 ± 10.32 years (Figure 1). All patients used arteriovenous fistula as vascular access for hemodialysis. The blood flow used in the sessions was 300 to 350 mL/min. Two patients were excluded due to limb amputation and weighing more than 100 kg.

Regarding clinical and laboratory characteristics, DW, height, V, BSA, Kt, Kt/BSA and V/BSA presented significant differences in the comparison between males and females (Table 1). Age and height presented significant differences in the comparison between group I (< 65 years) and group II (≥ 65 years) (Table 2).

**Table 1:** Clinical and laboratory characteristics of the study sample according to sex

	MALE (N = 31)	FEMALE (N = 16)	P
**Age**	63.61 ± 12.91	63.06 ± 8.39	= 0.878
**DW (kg)**	75.69 ± 14.81	64.75 ± 9.20	= 0.000*
**Height (m)**	1.69 ± 0.08	1.58 ± 0.10	= 0.000*
**BSA (m^2^)**	1.89 ± 0.22	1.70 ± 0.16	= 0.003*
**V (l)**	34.67 ± 4.21	30.82 ± 3.08	= 0.002*
**Albumin (g/dL)**	3.91 ± 0.33	3.86 ± 0.30	= 0.662
**Hemoglobin (g/dL)**	11.10 ± 1.74	11.21 ± 1.59	= 0.825
**KtVII**	1.20 ± 0.23	1.32 ± 0.15	= 0.076
**KtVOL**	1.32 ± 0.21	1.40 ± 0.30	= 0.291
**Kt**	41.61 ± 5.05	36.98 ± 3.70	= 0.002*
**KtBSA**	21.95 ± 0.16	21.74 ± 0.17	= 0.000*
**VBSA**	18.29 ± 0.16	18.12 ± 0.17	= 0.000*

* Significance by Student's t test.

**Table 2:** Clinical and laboratory characteristics of the study sample according to age

	GROUP 1 < 65 years	GROUP II ≥ 65 years	P
	(N = 27)	(N = 20)
**Age**	56.74 ± 9.65	72.45 ± 6.52	= 0.000*
**DW (kg)**	74.53 ± 15.01	68.50 ± 12.21	= 0.148
**Height (m)**	1.68 ± 0.10	1.62 ± 0.95	= 0.030*
**BSA (m^2^)**	1.87 ± 0.23	1.76 ± 0.19	= 0.088
**V (l)**	34.29 ± 4.53	32.11 ± 3.57	= 0.081
**Albumin (g/dL)**	3.89 ± 0.37	3.90 ± 0.32	= 0.979
**Hemoglobin (g/dL)**	10.82 ± 1.48	11.55 ± 1.86	= 0.142
**KtVII**	1.22 ± 0.23	1.27 ± 0.19	= 0.422
**KtVOL**	1.31 ± 0.26	1.41 ± 0.20	= 0.182
**Kt**	41.15 ± 5.44	38.53 ± 4.28	= 0.081
**KtBSA**	21.92 ± 0.18	21.82 ± 0.19	= 0.077
**VBSA**	18.27 ± 0.15	18.19 ± 0.16	= 0.077

* Significance by Student's t test.

**Figure 2:**
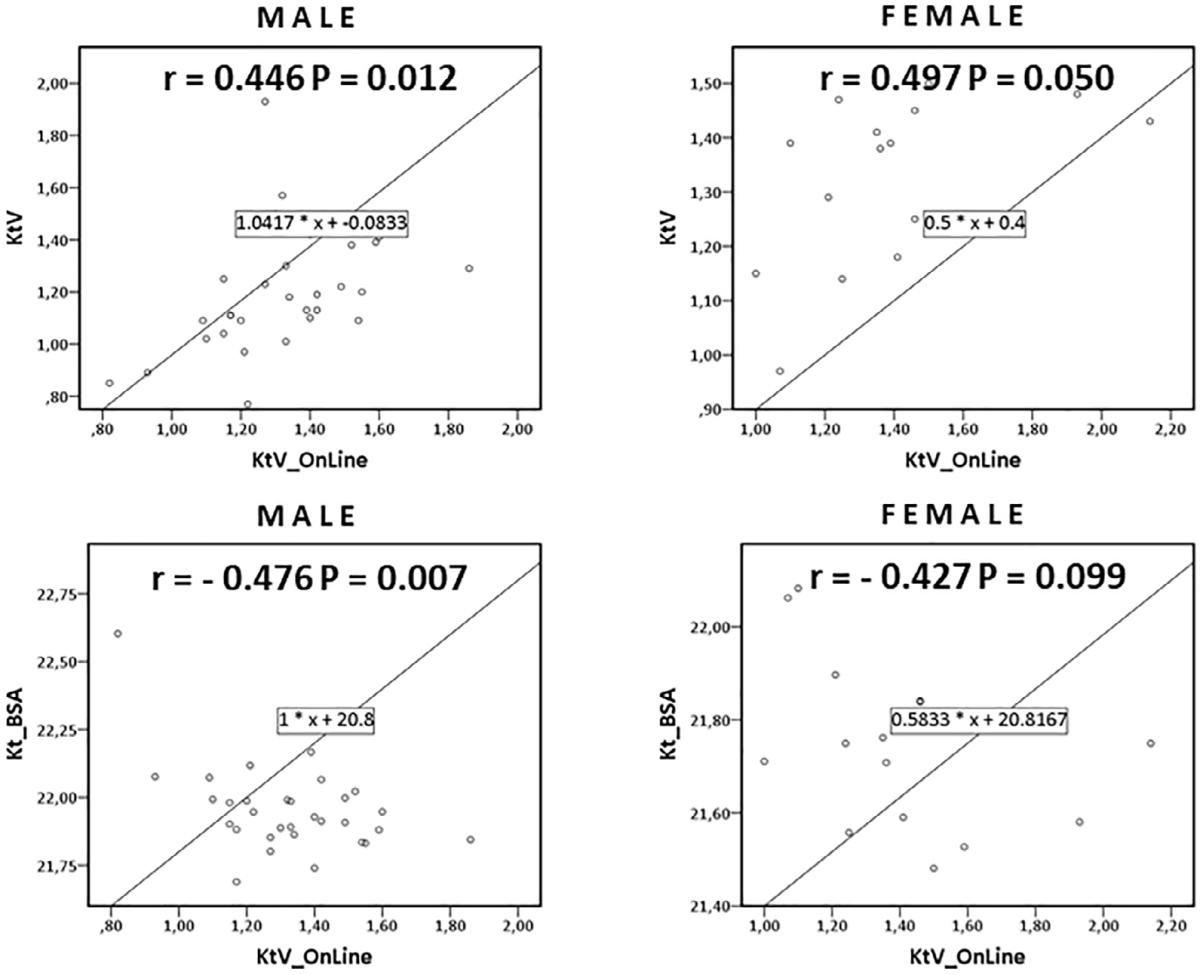
Pearson correlation scatter plot of Kt/VOL versus Kt/VII and Kt/VOL versus Kt/BSA according to sex.

**Figure 3:**
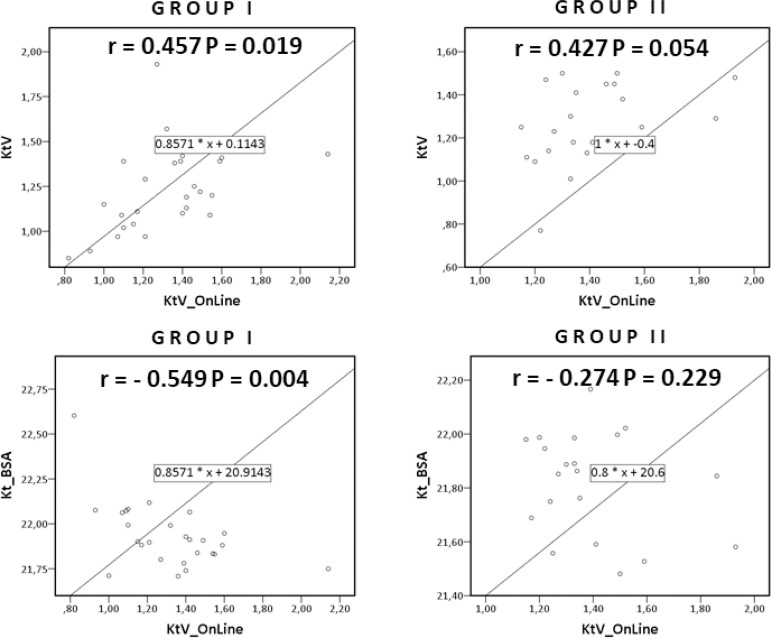
Pearson correlation scatter plot of Kt/VOL versus Kt/VII and Kt/VOL versus Kt/BSA according to age.

The appraisal of Kt/VII, Kt/VOL, and Kt/BSA of the 47 patients showed a significant Pearson correlation between Kt/VOL versus Kt/VII (0.452 P = 0.001) and between Kt/VOL versus Kt/BSA (-0.462 P = 0.001). Pearson correlation between Kt/VOL versus Kt/VII and Kt/VOL versus Kt/BSA was significant for males (0.446 P = 0.012 and -0.476 P = 0.007) and for group I (0.457 P = 0.019 and -0.549 P = 0.004), but not for females and group II (Figures 2 and 3). There was no agreement between Kt/VII versus Kt/BSA.

Men and group II received a larger dialysis dose than, respectively, females and group I, in the comparison between Kt/VOL versus Kt/VII (Table 3). Men and group I showed a relatively larger V/BSA than women and group II (Tables 4 e 5).

**Table 3:** Comparison of Kt/VOL versus Kt/VII

	KtVOL	KtVII	P
**TOTAL**	1.35 ± 0.24	1.24 ± 0.21	0.004*
**Male**	1.32 ± 0.21	1.20 ± 0.23	0.008*
**Female**	1.40 ± 0.30	1.32 ± 0.15	0.218
**Group I**	1.31 ± 0.27	1.23 ± 0.23	0.096
**Group II**	1.39 ± 0.20	1.26 ± 0.20	0.011*

* Significance by Student's t test.

**Table 4:** V/BSA ratio according to sex

	MALE	FEMALE	P
**V/BSA**	18.29 ± 0.13	18.12 ± 0.15	0.000

* Significance by Student's t test

**Table 5:** V/BSA ratio according to sex

	Group I	Group II	P
**V/BSA**	18.28 ± 0.15	18.18 ± 0.16	0.000

* Significance by Student's t test.

## Discussion

The Brazilian population on dialysis, as well as around the world, is aging^15^^,^ 16 ^,^ 17. A better understanding of the physiological differences and consequent needs in dialysis of this sub-population is necessary. The current clinical practice guidelines recognize that the effectiveness of dialysis varies between patients because of differences in body size and age, etc., so different people need different amounts of dialysis; the guidelines also inform on what defines “enough” dialysis and how to make sure each person is getting what he or she needs^18^. Older adults, often with associated frailty, usually have more adverse symptoms during hemodialysis^19^. At the same time, it is recognized that there is no survival benefit in octogenarians and nonagenarians with prolonged treatment time in hemodialysis^20^.

This is the first study in our knowledge that used the Kt/VOL through the ultraviolet absorbance in the spent dialysate to circumvent inaccuracies associated with age in the urea distribution volume estimate. Our study evidenced that males and individuals ≥ 65 years have a significant positive correlation between Kt/VOL and Kt/VII and a significant negative correlation between Kt/VOL and Kt/BSA. We did not observe significant correlations for females and individuals < 65 years. We also showed that males and individuals ≥ 65 years received a larger dialysis dose since Kt/VOL was larger than Kt/VII calculated for the same groups. Lastly, V/BSA ratio was significantly larger for men than women and individuals < 65 years than individuals ≥ 65 years. 

Real-time determination of Kt/V can be provided by monitoring ultraviolet light (UV) absorbance of solutes in spent dialysate in a specific wave-length^21^^,^ 22. High-performance liquid chromatography studies reported that many substances present in the uremic serum are active in the UV range of the light spectra^23^. Monitoring UV-absorbing compounds in spent dialysate not only offers enough data to tightly control a dialysis treatment but also eliminates the need for V, by directly obtaining the ratio K/V from the decaying absorbance curve. Castellarnau et al. suggest that UV absorbance is sufficiently representative of urea concentration so that more specific measurement of urea may not be necessary. The UV absorbance was less sensitive to measurement errors than the blood-sampling-based procedure^24^. Kt/VOL allows recognition of real-time dialysis urea kinetics regardless of sex^7^. Assuring that current standards are enough for all dialysis subpopulations should be a high priority goal for future quality improvement efforts.

Hemodialysis therapy is commonly scaled to V. The choice of V is governed by the fact that urea, which is distributed in body water, was initially chosen as the marker solute. Elimination of urea by dialysis follows first-order kinetics with an elimination constant equal to Kt/V, where K is the dialyzer clearance and V is the urea distribution volume, approximately equal to total body water content. The product of Kt/V, which can be considered a measure of dialysis intensity, and t, the dialysis session length (time), has been accepted as a measure of dialysis dose independent of body size. Concern has been raised about the relatively low dialysis dose (when expressed as Kt or liters of plasma cleared) provided to smaller patients and women^5^. These patients are known to have relatively low V values compared to their body surface area^6^^,^ 7. On the other hand, there is a risk of an inadequate dose of dialysis being offered to hemodialysis patients with high Watson volume. BSA is related to resting metabolic rate and correlates with body composition in both sexes. Normalizing glomerular filtration rate to BSA is standard practice. Normalizing dialyzer clearance, in the same way, would seem to be a logical extrapolation. Lowrie el al. reported that Kt/BSA is significantly associated with death risk, patients with a low Kt/BSA having an increased hazard ratio^25^.

The V used in the calculation of the dialysis dose has commonly been predicted from the limited, out-of-date Watson equations, based on nonrepresentative samples. Basile et al. reported that anthropometric equations for the estimation of V can be used only within a specific population to assess individual differences; they cannot be used to compare two different communities^26^. On the other hand, aging in the elderly is usually characterized by loss of fat-free mass, reduction in basal metabolic rate, and reduction of body water^27^. Thus, we can suggest that the normalization of Kt to V obtained by Watson’s formula would not be reliable in the elderly.

Use of BSA as the normalizing factor for hemodialysis prescription may represent a more physiological starting point, but many issues need to be refined to validate the concept before it can be used in the clinical arena. BSA relate only to basal metabolic requirements, while the generation of metabolic waste must relate to total energy expenditure. Total energy expenditure consists of resting energy expenditure plus physical activity energy expenditure plus the thermic effect of food^28^.

On the other hand, evidence highlight the contribution of the visceral organ mass, estimated by whole-body magnetic resonance imaging, to resting energy metabolism and uremic toxin production. Sarkar SR et al. investigated the association between small body mass index and increased mortality in chronic hemodialysis patients through analysis of body composition^29^. The difference between body mass and the sum of muscle, bone, and subcutaneous and visceral adipose tissue masses, measured by whole body magnetic resonance imaging, was defined as the high metabolic rate compartment representing the visceral mass. The authors found that a high metabolic rate compartment expressed in percent of body weight was inversely related to body weight and body mass index. In a multiple linear regression model, protein catabolic rate was significantly correlated only with high metabolic rate compartment. Consequently, uremic toxin production rate may be relatively higher in patients with low body weight and low body mass index as compared to their heavier counterparts. The poorer survival observed in smaller dialysis patients may be related to these relative differences. In line with this reasoning, the body surface would not be the ideal normalization for the dialysis dose. The Kt/VOL through the ultraviolet absorbance in the spent dialysate can correctly recognize a relatively higher urea production in patients with low BSA or contrariwise. 

By using Kt/VOL through the ultraviolet absorbance in the spent dialysate, our results reproduce the findings of the HEMO^5^ study of women receiving a lower dose of dialysis than men. At the same time, the authors indicate that patients over 65 years of age likewise receive a higher dose of dialysis than that offered to patients under 65 years of age. Both findings can only be justified by the fact that V does not represent the total amount of small molecules being removed, which is corroborated by our findings that no agreement was found between Kt/VOL versus Kt/VII and Kt/VOL versus BSA between women and patients over 65 years of age. Our findings can, in part, be explained by a higher V/BSA ratio for men than for women, the same way that it is higher for patients over 65 years than for those under 65 years.

The study was carried out with a small number of patients, which may have resulted in a moderate r in the correlations studied. Considerable variability is observed among different hemodialysis sessions about the time required to achieve a target dialysis dose, even when the same hemodialysis characteristics are maintained. Therefore, the dialysis dose achieved in one hemodialysis session is not necessarily representative of the Kt/V obtained in the other dialysis sessions^30^. Therefore, the study is open to criticism because it was carried out with data from a single hemodialysis session. Finally, we could have made the correlation with additional information such as the protein catabolism rate to enhance the clinical applicability of the Kt/VOL through the ultraviolet absorbance in the spent dialysate.

In conclusion, the results of our study suggest that people over 65 receive a proportionally higher dose of dialysis than those under that age when evaluated by a more reliable method such as Kt/V OnLine through ultraviolet absorbance in the dialysate. Kt/V OnLine corrects inaccuracies associated with the estimation of V and is considered more reliable than the usual calculation for urea kinetics^31^. Further work is required to develop these concepts and to translate them into rigorous outcome-based adequacy targets suitable for clinical usage.
